# In Vitro and In Vivo Effects of the Urokinase Plasminogen Activator Inhibitor WX-340 on Anaplastic Thyroid Cancer Cell Lines

**DOI:** 10.3390/ijms23073724

**Published:** 2022-03-28

**Authors:** Enke Baldini, Dario Presutti, Pasqualino Favoriti, Simonetta Santini, Giuliana Papoff, Chiara Tuccilli, Raffaella Carletti, Cira Di Gioia, Eleonora Lori, Iulia Catalina Ferent, Federica Gagliardi, Antonio Catania, Daniele Pironi, Domenico Tripodi, Vito D’Andrea, Salvatore Sorrenti, Giovina Ruberti, Salvatore Ulisse

**Affiliations:** 1 Department of Surgical Sciences, “Sapienza” University of Rome, 00161 Rome, Italy; enke.baldini@uniroma1.it (E.B.); pasqualino.favoriti@gmail.com (P.F.); chiara.tuccilli@gmail.com (C.T.); eleonora.lori@uniroma1.it (E.L.); iulia.ferent@uniroma1.it (I.C.F.); federica.gagliardi@uniroma1.it (F.G.); antonio.catania@uniroma1.it (A.C.); daniele.pironi@uniroma1.it (D.P.); domenico.tripodi@uniroma1.it (D.T.); vito.dandrea@uniroma1.it (V.D.); salvatore.sorrenti@uniroma1.it (S.S.); 2 Institute of Biochemistry and Cell Biology (IBBC), National Research Council (CNR), Monterotondo, 00015 Rome, Italy; dpresutti@ichf.edu.pl (D.P.); santini.simonetta80@gmail.com (S.S.); giuliana.papoff@cnr.it (G.P.); giovina.ruberti@cnr.it (G.R.); 3 Department of Radiological, Oncological and Pathological Sciences, “Sapienza” University of Rome, 00161 Rome, Italy; raffaella.carletti@uniroma1.it (R.C.); cira.digioia@uniroma1.it (C.D.G.)

**Keywords:** anaplastic thyroid cancer, urokinase plasminogen activator, WX-340, xenograft, proliferation, cell adhesion, cell migration, BHT-101, CAL-62

## Abstract

Increased expression of the urokinase-type plasminogen activator (uPA) system is associated with tumor invasion, neo-angiogenesis, and metastatic spread, and has been shown to positively correlate with a poor prognosis in several cancer types, including thyroid carcinomas. In recent years, several uPA inhibitors were found to have anticancer effects in preclinical studies and in some phase II clinical trials, which prompted us to evaluate uPA as a potential therapeutic target for the treatment of patients affected by the most aggressive form of thyroid cancer, the anaplastic thyroid carcinoma (ATC). In this study, we evaluated the in vitro and in vivo effects of WX-340, a highly specific and selective uPA inhibitor, on two ATC-derived cell lines, CAL-62 and BHT-101. The results obtained indicated that WX-340 was able to reduce cell adhesion and invasiveness in a dose-dependent manner in both cell lines. In addition, WX-340 increased uPA receptor (uPAR) protein levels without affecting its plasma membrane concentration. However, this compound was unable to significantly reduce ATC growth in a xenograft model, indicating that uPA inhibition alone may not have the expected therapeutic effects.

## 1. Introduction

Epithelial thyroid cancer (TC) represents about 96% of all endocrine malignancies and is one of the most frequent cancers in women [1,2,3]. Its incidence has increased over the last few decades, mainly as a result of the improved ability to diagnose malignant transformation in small non-palpable thyroid nodules [3,4,5]. Grounded on histological and clinical behavior, TC are divided in well-differentiated TC (DTC), comprising the papillary (PTC) and follicular (FTC) histotypes, poorly differentiated TC (PDTC), and undifferentiated or anaplastic TC (ATC) [6]. The PTC has a propensity to spread via lymphatic vessels to local lymph nodes, while FTC is characterized by hematogenous diffusion producing lung and bone metastases [6]. The less differentiated and more aggressive PDTC and ATC arise from the dedifferentiation of DTC, as suggested by the frequently encountered coexistence of DTC with PDTC or ATC bearing similar genetic alterations [6,7,8,9,10,11]. The PDTC was included as a separate entity in the WHO classification of TC in 2004, and it is defined as a thyroglobulin producing, non-follicular, and non-papillary TC, having an intermediate clinical behavior between DTC and ATC, characterized by widely infiltrative growth, necrosis, vascular invasion, and numerous mitotic figures [8,11]. The ATC appear as disseminated fleshy masses in the neck with areas of necrosis and hemorrhage. They are composed of undifferentiated cells negative for common thyroid-lineage markers (e.g., thyroglobulin and natrium-iodide symporter), and originate three morphological patterns: squamoid, pleomorphic giant cells, and spindle cells [6]. 

Total thyroidectomy followed by adjuvant therapy with ^131^I is the treatment of choice for most patients affected by DTC, resulting in a 10-year survival rate of around 90% [12,13]. Comparatively, adverse outcomes are usually observed in patients with PDTC and ATC, in which the reduced expression of the natrium/iodide symporter (NIS) renders the ^131^I treatment ineffective, and external-beam irradiation or chemotherapy are equally unsuccessful [14,15,16]. Patients with ATC have the worst prognosis with an average survival time of a few months from diagnosis, and are little or unaffected by current cancer treatments. These patients present with a rapidly growing thyroid mass and locoregional symptoms (i.e., dyspnea, dysphagia, neck pain, hoarseness, and stroke), and in the majority of cases death occurs following tumor airway obstruction [17]. Therefore, the identification of new therapeutic approaches capable of ameliorating the fate of PDTC and ATC patients is sorely needed. 

The urokinase plasminogen activator (uPA) system (uPAS) consists of the uPA, its cognate receptor (uPAR) and two specific inhibitors, the plasminogen activator inhibitor 1 (PAI-1) and 2 (PAI-2) [18]. The uPA converts the proenzyme plasminogen in the serine protease plasmin, involved in a number of physiopathological processes requiring basement membrane (BM) and extracellular matrix (ECM) remodeling, including tumor progression and metastatization [18]. Data accumulated over the past years have made it increasingly clear that the uPAS has a multifunctional task in the neoplastic evolution, affecting tumor angiogenesis, malignant cell proliferation, adhesion, migration, intravasation, and growth at the metastatic site. Their relevance in cancer progression is supported by the finding of an increased expression of uPA, uPAR, and PAI-1 in several malignant tumors, and by the positive correlation between levels of one or more uPAS members and a poor prognosis [18]. Over the last two decades, independent studies investigating the expression of uPAS components in normal and cancer thyroid cells and tissues described alterations of uPAS components following malignant transformation [18,19]. More importantly, the upregulation of uPA, uPAR, and PAI-1 was associated with high-risk clinicopathological factors, such as lymph node metastasis, higher TNM stage, shorter disease-free interval, and overall survival [19,20,21,22,23,24,25]. 

To date, little information exists specifically concerning the expression of uPAS components in PDTC or ATC, but data produced so far by independent studies agree in reporting a trend of increased expression of uPA, uPAR, and PAI-1 in these aggressive forms compared to DTC [20,26,27]. As for other types of solid tumors, experimental findings suggest that uPAS components could represent valuable therapeutic targets also in thyroid cancers [18,28,29]. Inhibition can be implemented at multiple levels, i.e., by impairing uPA and uPAR gene expression, uPA catalytic activity, uPA–uPAR binding, or uPAR interaction with downstream effectors [18,30]. Over the years, several anti-uPA and anti-uPAR compounds have been developed and tested both in vitro and in animal tumor models, some of which showed effectiveness in preclinical studies and also in phase I/II clinical trials [18,30]. However, current knowledge on the therapeutic efficacy of uPA–uPAR inhibitors on PDTC and ATC is still very limited. Anticancer effects on ATC cells were observed both in vivo and in vitro by inhibiting transcription factors that promote uPA expression, i.e., NF-κB and FOXM1, but multiple genes are regulated by these factors and thus the final result could be due to the simultaneous dampening of several pathways [31,32]. More direct evidence emerged from a recent study showing that infection of ATC cells with a urokinase-targeted recombinant oncolytic Sendai virus induced uPA-dependent cell fusion and cytotoxicity, and had remarkable therapeutic efficacy in orthotopic ATC mouse models [33]. 

On this basis, our study was aimed at exploring the effects of a small molecule uPA inhibitor (WX-340) on the tumorigenic behavior of two human cell lines derived from ATC by means of in vitro and in vivo experiments.

## 2. Results

**Figure 1:**
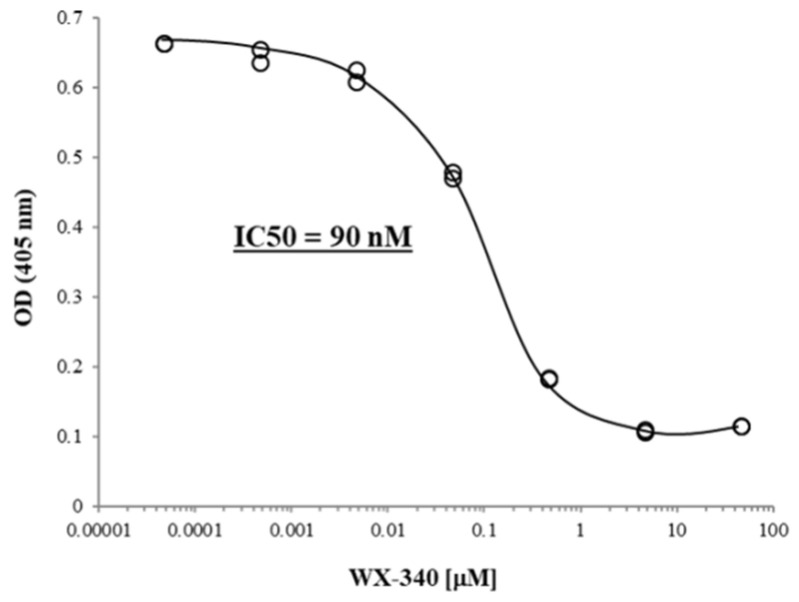
uPA inhibition assay. The activity of 10 Units of uPA purified from human urine was measured with increasing concentrations of WX-340 (50 pM to 50 μM). IC50: half-maximal inhibitory concentration.

Initially, the inhibitory effect of WX-340 was monitored in vitro on uPA isolated from human urine by means of colorimetric assay. A concentration range of 50 pM to 50 μM was used to inhibit 10 Units of uPA, and the resulting IC50 was 90 nM (Figure 1).

**Figure 2:**
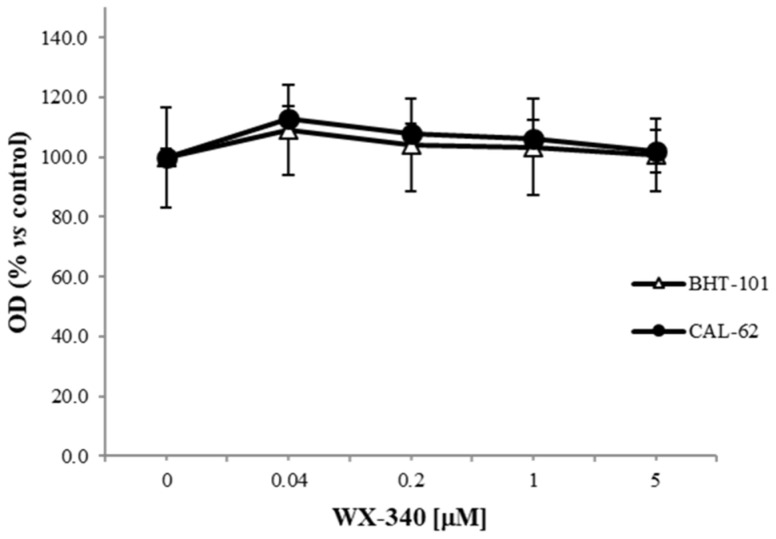
Dose-response proliferation assay. Cells were treated with increasing doses of WX-340 for 3 days, after which the tetrazolium salt WST-1 was added to cultures, and production of formazan dye was read after 2 h by microplate reader. The average absorbance value of each point at T = 72 h was subtracted to the average value measured in a plate at T = 0 h.

The growth of the ATC-derived cell lines CAL-62 and BHT-101 in the presence of WX-340 was tested in dose-response experiments at two incubation times. No variations in the mitochondrial dehydrogenases activity, directly proportional to the number of living cells, was recorded in treated cells compared to control cells after 24 h and 72 h (see Figure 2). Neither WX-340-dependent cell fusion nor cytotoxicity were observed in cell cultures.

**Figure 3:**
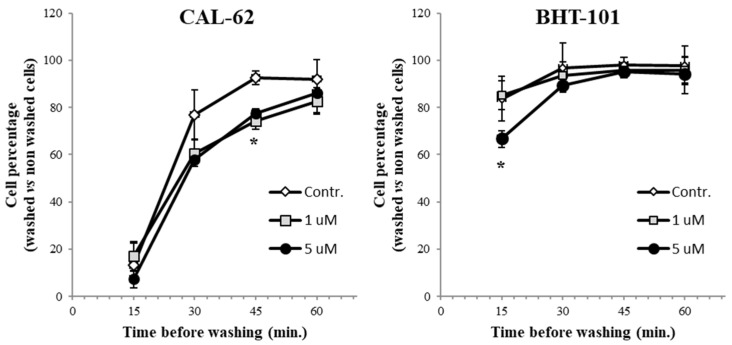
Adhesion assay. Cells pre-treated with increasing doses of WX-340 for 24 h were detached with trypsin/EDTA, counted, and seeded in a 96-well plate. Separate well rows were washed after 15 min, 30 min, 60 min, or not washed. Finally, WST-1 was added to all wells and the OD was measured 2 h later. * *p* < 0.05.

Then, we tested the ability of WX-340 to interfere with ATC cell adhesion by putting cells pre-treated or not with the drug in a 96-well plate, and washing individual rows of wells at regular time points. A significant delay in adhesion of CAL-62 cells was achieved at the 1 μM dose, while for BHT-101 a similar delay was obtained with the 5 μM dose (see Figure 3). Further increases in drug quantity did not exert greater effects on both cell lines.

**Figure 4:**
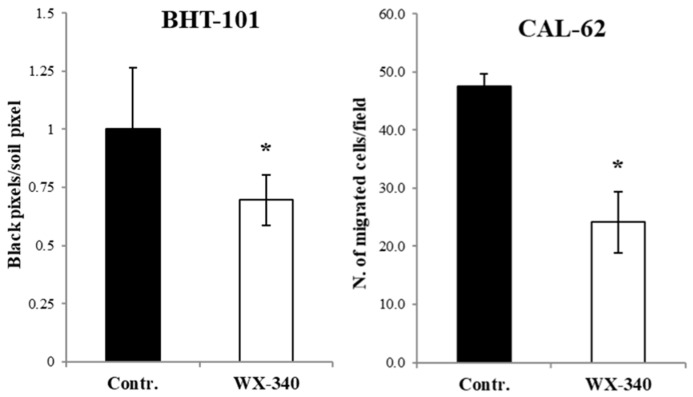
Invasion assay through Matrigel-coated nucleopore membranes. Cells pre-treated or not with WX-340 5 μM for 24 h were counted and suspended in serum-free medium with or without fresh added WX-340, then seeded in the upper reservoirs of Boyden chambers. The **lower** reservoirs were filled with medium containing FBS as chemoattractant. After 6 h incubation, nucleopore membranes were fixed and stained with violet Cresyl solution. Non-migrated cells were removed from the upper layer, and migrated cells were estimated by cell count (CAL-62) or by measuring the ratio black pixels/white pixels in binarized photos of membranes with the ImageJ software 1.53 (BHT-101). * *p* < 0.05.

We also evaluated the invasive behavior of ATC cells incubated in culture medium containing the drug before and during the assay. The results obtained demonstrated that the presence of WX-340 significantly reduced cell migration across an ECM layer by about 50% for CAL-62 and 30% for BHT-101 compared to controls (see Figure 4).

**Figure 5:**
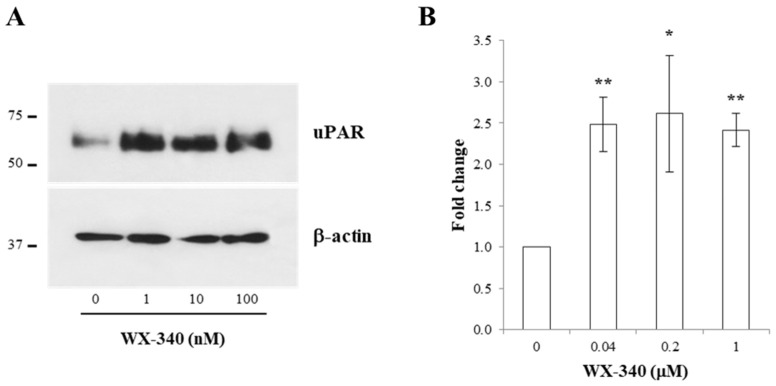
(**A**) Representative Western blotting experiment with BHT-101 treated with increasing doses of WX-340; (**B**) densitometric analysis of the immunoblotted bands. * *p* < 0.05; ** *p* < 0.01.

Moreover, we verified if the functional impairment of the uPA–uPAR complex could lead to changes in either the whole-cell amount of uPAR or its plasma membrane fraction. From Western blotting experiments with total extracts of treated and non-treated BHT-101 cells, a more than 2-fold increase in uPAR signal was observed at the dose 1 nM, much lower than those producing functional effects (Figure 5A,B). 

**Figure 6:**
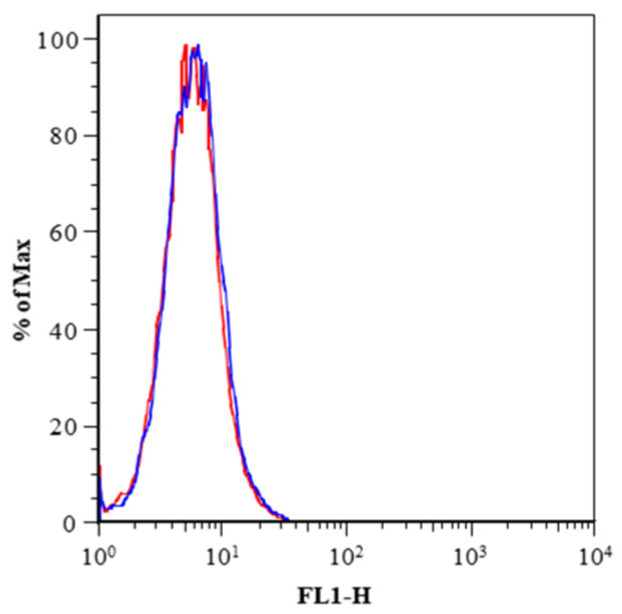
Citofluorimetric detection of membrane uPAR in BHT-101 cells treated with WX-340 compared to control cells.

However, a concomitant increase in the share of membrane uPAR, measured by citofluorimetric analysis, was not observed even at 5 μM (Figure 6).

**Figure 7:**
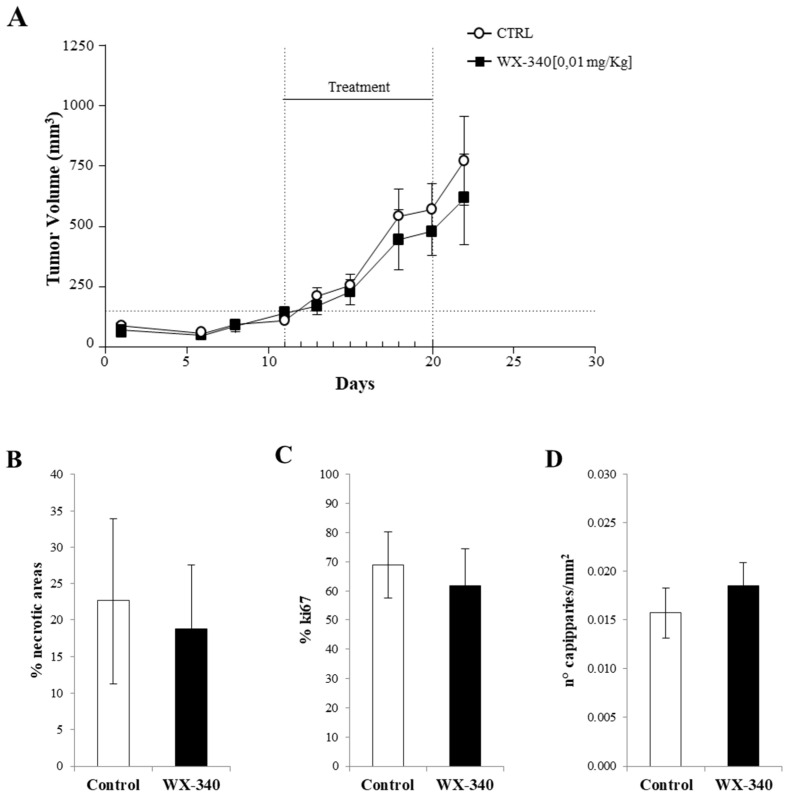
(**A**) Growth curves of tumor masses in ATC xenograft models. For convenience, only one group of treated mice is represented together with the control group. Quantitative analysis of necrosis (**B**), proliferation index (**C**), and vessel density (**D**) in tumor sections of treated mice vs. control mice. Results are expressed as mean ± SD.

Finally, we tested the in vivo efficacy of WX-340 in counteracting tumor growth in a xenograft mouse model of ATC obtained with the BHT-101 cell line. To this end, we worked with five groups of athymic nude mice, four of which received the drug in different doses and the fifth the vehicle alone. A slight reduction in nodule size was observed in treated mice, however no statistically significant reductions in final tumor masses were found in any of the drug-treated groups. A representative example of these results is shown in Figure 7A. We also examined tumor sections by IHC experiments in order to observe eventual structural changes produced by the drug. Again, there were no appreciable differences in proliferation, percentage of necrotic tissue, and capillary density (see Figure 7B–D).

## 3. Discussion

To our knowledge, there is currently very little information about the expression profile of uPAS components in ATC. However, the uPAS is overexpressed in the majority of DTC and associated with high-risk clinicopathological features, thus it is reasonable to believe that it also comes into play significantly in ATC. 

In our experimental setting, we observed an increase in the total cellular amount of uPAR protein following uPA inhibition, which could be interpreted as an enhanced gene expression and a slowdown in receptor turnover aimed at compensating for the poor activity of the uPA–uPAR complex. However, the plasma membrane fraction of uPAR appeared unchanged, and this is a somewhat unexpected result, which will require further investigation to be explained. As a consequence of the impaired uPA activity, ATC cells showed reduced invasive capacity in vitro, and a slight but significant delay in the formation of new cell adhesions. Contrarily from what was reported by Miyagawa and colleagues [33], we did not notice induction of cell death nor fusion in treated cultures. Although these authors used different ATC cell lines, this discrepancy is presumably due to the different mode of uPA blockade, i.e., viral infection of cells and subsequent protein cleavage rather than competitive inhibition by a small molecule. Furthermore, the most substantial difference between Miyagawa’s study and ours concerns the in vivo experiments, because the administration of WX-340 has not been proved effective in counteracting tumor growth in xenografts. It can be assumed that the uPA-targeted oncolytic virus allows a more capillary and homogeneous inhibitory effect within tumor masses compared to the passive diffusion of a chemical compound. Secondly, it is noteworthy that while some uPA or uPAR inhibitors have been found capable of producing cytotoxic damage and decreased proliferation in some tumor cell types, induction of cell fusion was achieved only with this virotherapy approach, and it is most likely caused by the viral infection itself regardless of the uPA cleavage. This effect, combined with the intrinsic cytotoxicity recognized for the control viral vector, is supposed to contribute considerably to the observed antitumor effect [33].

On the whole, our results indicate that uPA inhibition alone is not sufficient to halt the neoplastic growth of ATC. In view of the complexity and multiplicity of functions performed by the uPAS, several hypotheses could be advanced to explain this failure. The uPA-dependent proteolytic action of the plasmin could be partially compensated by that of matrix metalloproteinases (MMPs), some of which are upregulated in ATC [34,35,36]. On the other hand, some uPAR functions do not rely on uPA enzymatic activity, and are mediated by the binding of the receptor with extracellular matrix components (i.e., vitronectin) or membrane proteins (i.e., integrins, members of the low-density lipoprotein receptor family, tyrosine kinase receptors, and G protein-coupled receptors) [18,37]. These interactions trigger various intracellular signaling cascades affecting cell proliferation, adhesion, and motility. In addition, proteolytic cleavage of the membrane uPAR releases soluble uPAR (suPAR), including the entire extracellular moiety, or smaller fragments. These uPAR forms are endowed with biological activities such as cell migration, chemotaxis, and uPA scavenging, and can interfere with cellular uPAR functions [37,38].

Despite such potential shortcoming referable to all cancers, small molecule uPA inhibitors were shown to produce significant antineoplastic effects with other types of solid tumors [39,40,41], which suggests the ability of ATC cells to compensate for the uPAS functions turned off by drug treatment and to activate resistance mechanism(s). Although a negative outcome is obviously disappointing when testing a potential therapeutic agent, we believe that the information provided by this work may turn useful in a research area where it is still necessary to increase efforts aimed at identifying an effective therapeutic approach for the treatment of a highly aggressive and lethal cancer.

## 4. Materials and Methods

### 4.1. uPA Inhibitor

WX-340 is a small molecule competitive active-site inhibitor of the uPA enzymatic activity with high potency and specificity, kindly provided to us by Wilex (Munich, Germany) under Material Transfer Agreement. This compound was shown to inhibit human uPA with Ki = 0.012 µM, and mouse uPA with Ki = 0.170 µM, while for other serine proteases (i.e., human plasmin, thrombin, tissue-PA, and factor Xa), the Ki were greater than 190 µM [34]. WX-340 was dissolved in D-mannitol 5% (*w*/*v*) aqueous solution, and stored in aliquots at −20 °C until use. Before starting the experiments, the in vitro activity was tested with the CHEMICON uPA Activity Assay Kit (Merck Life Science, Darmstadt, Germany), and the IC50 was calculated by means of the GraphPad Prism software 6 (GraphPad Software, La Jolla, CA, USA).

### 4.2. Cell Cultures

The ATC-derived cell lines BHT-101 and CAL-62 were purchased from DSMZ (Braunschweig, Germany). Cell cultures were maintained in medium DMEM containing 10% (CAL-62) or 20% (BHT-101) fetal bovine serum (FBS) and 2 mM L-glutamine, at 37 °C in 5% CO_2_ humidified atmosphere. In all the experiments, controls were performed by treating cells with the drug diluent at the same volume of the highest dose of WX-340 administered. Culture media with or without inhibitor were changed every day until the end of the incubation time.

### 4.3. Proliferation Assay

Cells were seeded in 96-well plates (2.000 cells/well). After 4 h, the cell proliferation reagent WST-1 was added to one plate, and the absorbance was measured 2 h later (T = 0) with a microplate ELISA reader (Tecan Group Ltd., Männedorf, Switzerland). Other plates were treated with increasing doses of WX-340 (from 0 to 5 μM) for one day (T = 24) or 3 days (T = 72), after which the WST-1 was added, and the absorbance read after 2 h. The average absorbance value of each point at T = 24 h and at T = 72 h was subtracted to the average value measured in a plate at T = 0 h. 

### 4.4. Adhesion Assay

Cells cultured in Petri dishes were treated with increasing doses of WX-340 and incubated for 24 h. After that, the cells were detached with trypsin/EDTA, counted, resuspended, and put in a 96-well plate (8000 cells/well). Individual rows of wells were washed 3 times with complete medium after 15 min, 30 min, or 60 min, while control wells were not washed. Upon the last wash, WST-1 was added to all wells and the absorbance was measured 2 h later. The average absorbance value of washed rows at each time lapse was expressed as a percentage of the average value measured for non-washed rows.

### 4.5. Invasion Assay

Whatman nuclepore polycarbonate hydrophilic membranes with 8 μm pore size (Cytiva, Loughborough, Leicestershire, UK) were put into Boyden chambers and pre-coated by laying on top 25 μg of ECM Gel from Engelbreth-Holm-Swarm murine sarcoma (Merck, Darmstadt, Germany), and letting it solidify overnight. Adherent cells were cultured in complete medium supplemented with WX-340 5 μM for 24 h. Thereafter, cells were detached with trypsin/EDTA, counted, and resuspended at a concentration of 500,000 cells/mL in serum-free medium containing 0.1% bovine serum albumin and WX-340 5 μM. The lower compartment of each chamber was filled with complete medium as chemoattractant, while in the upper one 0.5 mL of cell suspension was added. Controls were prepared in the same way without adding the drug. After 6 h incubation at 37 °C in 5% CO_2_ humidified atmosphere, the membranes were placed in a 12 well plate, fixed with 95% ethanol for 5 min and colored with violet Cresyl solution. The membranes were positioned on slides so as to have non-invasive cells on the upper side, which were removed by rubbing the surface with a cotton swab. Migrated CAL-62 cells were counted under a microscope in 5 fields for each membrane, but migrated BHT-101 cells tended to form clusters, making manual counting difficult. Hence, these membranes were photographed with a camera installed on an optical microscope, the grayscale pictures were binarized, and the ratio black pixels/white pixels—proportional to the cell density—was measured with the ImageJ software (NIH, Bethesda, MD, USA). 

### 4.6. Western Blotting

Cells were treated with increasing doses of WX-340 for 24 h, then lysed in RIPA buffer with fresh added protease inhibitors. A total of forty μg of proteins were run in SDS-PAGE under non-reducing conditions, and blotted onto nitrocellulose membranes. These were saturated for 2 h with 5% nonfat dry milk in TBST, then incubated overnight at +4 °C with primary antibodies anti-uPAR 1:2000 (R&D Systems, Minneapolis, MN, USA) and anti-β-actin 1:20,000 (Sigma-Aldrich, Milan, Italy). After washing, membranes were incubated with appropriate HRP-conjugated secondary antibodies 1:2000 (Thermo Fisher Scientific, Rockford, IL, USA), and developed using the LiteAblot EXTEND (for uPAR) or PLUS (for β-actin) chemiluminescent substrates (Euroclone, Milan, Italy). Densitometric analysis was performed with the ImageJ software.

### 4.7. Flow Cytometric Analysis

Cell cultures were treated with WX-340 5 μM or the diluent for 48 h, then harvested by scraping in cold phosphate buffered saline (PBS), washed, counted, and diluted in PBS. One-million cell suspensions were mixed to anti-uPAR antibody diluted 1:100 in PBS, and incubated on ice for 30 min, washed, and incubated with anti-mouse FITC-conjugated antibody diluted 1:50 in PBS for 30 min on ice in the dark. Negative controls were made in parallel by omitting the primary antibody or the secondary antibody, or both. After washing, cells were resuspended in PBS, and propidium iodide was added at a final concentration of 50 μg/mL per million cells. Flow-cytometric analysis was performed with the FACScalibur Flow cytometer and CellQUEST software 3.3 (BD Biosciences, San Jose, CA, USA). 

### 4.8. In Vivo Experiments

Athymic nude female mice (Foxn1nu/Foxn1+) (Harlan Laboratories) were housed in individually ventilated cages (IVC) under controlled conditions (20–22 °C; 55–65% relative humidity; 12/12 h light/dark cycle; standard irradiated diet and water ad libitum). At 5 weeks of age, the mice received a subcutaneous injection of 4.5 × 10^6^ BHT-101 cells in PBS. After two weeks, the tumor masses reached approximately 100 mm^3^, and mice were randomly divided into 5 groups of 5 animals each. The WX-340 was administered daily via intraperitoneal injection in a volume of 100 μL at the following doses: 10 mg/kg, 1 mg/kg, 0.1 mg/kg, and 0.01 mg/kg. An equal volume of diluent was administered to the control group. The dose regimen was chosen based on a previous experiment performed with the rat BN-472 mammary carcinoma model, slightly widening the WX-340 concentration range used in this work [42]. Tumor sizes were measured with a caliper every 2 days. When the tumor volumes reached an average of approximately 0.6–0.9 cm^3^, the mice, previously euthanized with intra-peritoneal injections of Tiletamine/Zolazepam (800 mg/kg) and Xylazine (100 mg/kg), were sacrificed and tumors were harvested and measured. Excised tumors were fixed with 3.7% paraformaldehyde (PFA) (*w*/*v*) 1× PBS for 20 min at room temperature and then embedded in paraffin. Consecutive tissue sections of 3 µm were cut and deparaffinized in xylene, rehydrated through graded alcohol series, and stained with hematoxylin–eosin (HE) for microscopic morphological analysis. Other sections were immunostained to detect the proliferation marker Ki67 and the endothelial marker CD31. At first, antigen retrieval was performed by putting the slides into a microwaves (750 W) in pH 6 citrate buffer 0.01 mol/L for 20 min. Sections were incubated for 1 h at room temperature with primary monoclonal antibodies anti-Ki67 (1:50, clone M7240, Dako, CA, USA) and anti-CD31 (1:100, clone JC70A, Dako, CA, USA). Endogenous peroxidase activity was blocked with 3% hydrogen peroxide for 15 min, then sections were incubated with secondary HRP-conjugated antibodies, and a positive immunoreaction was observed by adding of 3,3′-diaminobenzidine (DAB) and counterstaining with Mayer hematoxylin. The reaction was amplified by means of the Universal LSAB2 System-HRP (Dako, CA, USA). Negative control sections were prepared in parallel omitting the primary antibody. Pictures of HE- and immunostained sections were captured with the Aperio scanner (Leica Biosystems), and randomly selected images were analyzed to quantify necrotic tissue, proliferation index, and blood microvessel density using the ImageJ software. The necrotic tissue was evaluated as a percentage of necrotic areas relative to the total area, and the proliferation index was expressed as a percentage of Ki67 positive cells out of the total number. The blood microvessel density was calculated as ratio of the number of CD31+ vessels to the total area of five randomly selected fields per slide. Sections were viewed by two independent investigators unaware of the sample identity, using a Leica microscope (Leitz Camera, Wetzlar, Germany).

### 4.9. Statistics

All the results were obtained from three independent experiments, and are expressed as mean ± S.D. Dose-response curves were analyzed by fitting points with four-parameter logistic functions using the MyCurveFit program. Differences among groups were evaluated by the Mann–Whitney U test, using the SPSS software 25 (Armonk, NY, USA). Data were considered statistically different when the pertaining *p* values were <0.05.

## Data Availability

Data and information related to this study are available upon request.
